# Supplementary material for: Manifold learning analysis suggests strategies to align single-cell multimodal data of neuronal electrophysiology and transcriptomics
Source: Commun Biol. 2021 Nov 19;4:1308. doi: 10.1038/s42003-021-02807-6 (PMC8604989; doi:10.1038/s42003-021-02807-6)
Supplement: Supplementary file 2 — Supplementary Information [file 42003_2021_2807_MOESM2_ESM.pdf]

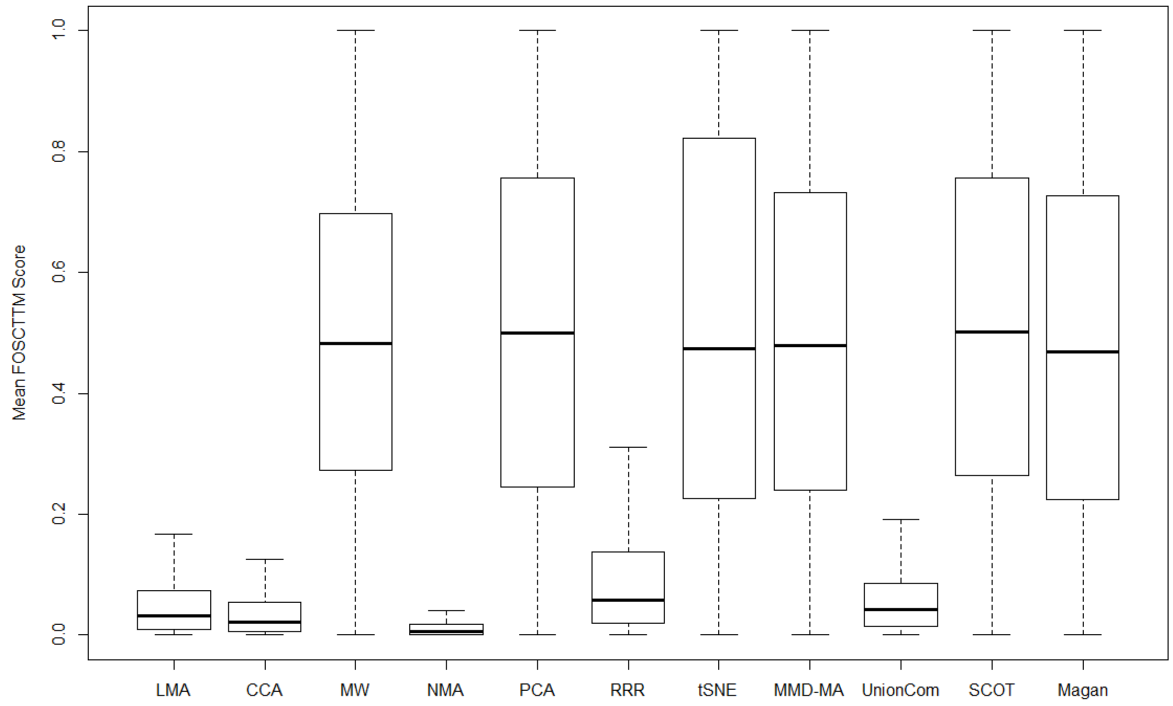

Figure S1 Boxplots show the pairwise cell Mean FOSCTTM Score after alignment on the latent space for 3654 neuronal cells (aspiny) in the mouse visual cortex (Methods). FOSCTTM: fractions of samples closer than the true match. The cell coordinates on the latent space are standardized per cell (i.e., each row of $\tilde{X} = [\tilde{X}_e, \tilde{X}_t]$) to compare methods. Each box represents one alignment method. The box indicates the lower and upper quantiles of the data, with a horizontal line at the median. The vertical line extended from the boxplot shows a 1.5 interquartile range beyond the 75th percentile or 25th percentile. The machine learning methods for alignment include linear manifold alignment (LMA), nonlinear manifold alignment (NMA), manifold warping (MW), Canonical Correlation Analysis (CCA), Reduced Rank Regression (RRR), Principal Component Analysis (PCA, no alignment), t-SNE (Stochastic Neighbor Embedding, no alignment), MMD-MA (Manifold Alignment with maximum mean discrepancy measurement), unsupervised topological alignment of single-cell multi-omics integration (UnionCom), Single-Cell alignment using Optimal Transport (SCOT), and Manifold Aligning GAN (MAGAN).

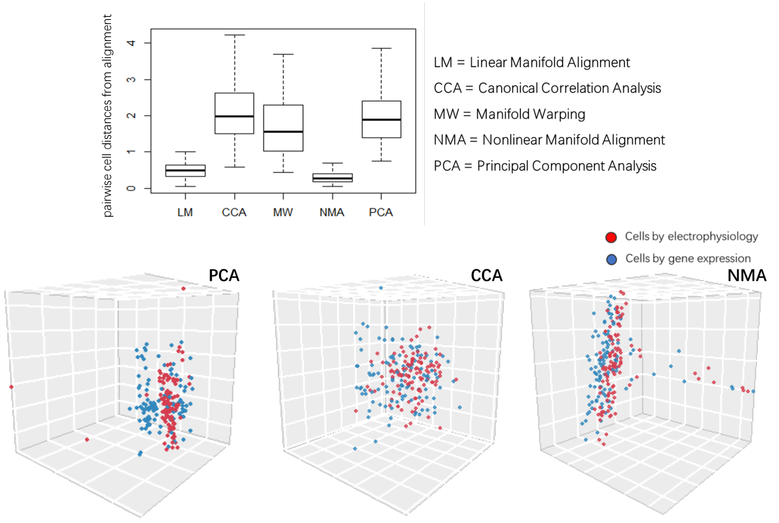

Figure S2 We also tested multiple machine learning approaches to align another small-scale Patch-seq dataset in the mouse visual cortex. This dataset includes 112 cells with electrophysiological data and gene expression data [12]. We used 100 most variable genes and 11 standardized e-features (excluding afterdepolarization and latency), suggested by the paper [12], and applied multiple state-of-the-arts Multiview learning methods to align the cells (Top boxplot). The nonlinear manifold alignment outperformed others, implying strong nonlinear relationships between cellular electrophysiology and gene expression. Such cross-modal relationships also suggest potential GRNs controlling gene expression driving other modalities. (Bottom) The 112 neuronal cells on the latent space (3D) after alignment by PCA (no alignment), CCA and NMA.

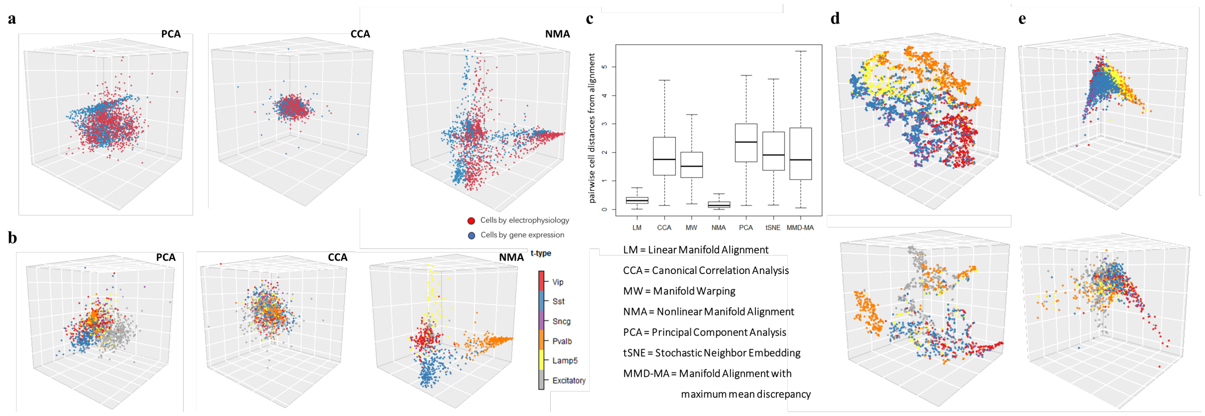

Figure S3 (**a**) The cells on the latent space (3D) after alignment of 1208 neuronal cells in the motor cortex by PCA (no alignment), CCA and NMA. The red and blue dots represent the cells from gene expression and electrophysiological data, respectively. The blue dots are drifted -0.05 on the y-axis for all 3 methods and x,y and z coordinates are flipped for NMA method to better visualize the trajectory. (**b**) Boxplots show the pairwise cell distance (Euclidean Distance)

after alignment on the latent space for 1208 neuronal cells in the mouse motor cortex while setting nearest neighbor to be 2 (Methods). The cell coordinates on the latent space are standardized per cell (i.e., each row of $\tilde{X} = [\tilde{X}_e, \tilde{X}_t]$) for comparison across methods. Each box represents one alignment method. The box indicates the lower and upper quantiles of the data, with a horizontal line at the median, the vertical line extended from the boxplot shows 1.5 interquartile range beyond the 75th percentile or 25th percentile. The machine learning methods for alignment include linear manifold alignment (LM), nonlinear manifold alignment (NMA), manifold warping (MW), Canonical Correlation Analysis (CCA), Principal Component Analysis (PCA, no alignment), t-SNE (Stochastic Neighbor Embedding) and MMD-MA (Manifold Alignment with maximum mean discrepancy measurement). (**c**) Scatterplots show 1208 neuronal cells in the mouse motor cortex from electrophysiological data on the latent spaces (3D) after alignment by PCA (no alignment), CCA and NMA. The cells are colored by prior known transcriptomic types (t-types), among which those get over 100 observations are drawn. Red: Vip type; Blue: Sst type; Purple: Sncg type; Orange: Pvalb type; Yellow: Lamp5 type; Grey: Excitatory. (**d**) t-SNE visualization of cells without alignment for the visual cortex (top) and the motor cortex (bottom). (**e**) RRR visualization of cells for the visual cortex (top) and the motor cortex (bottom).

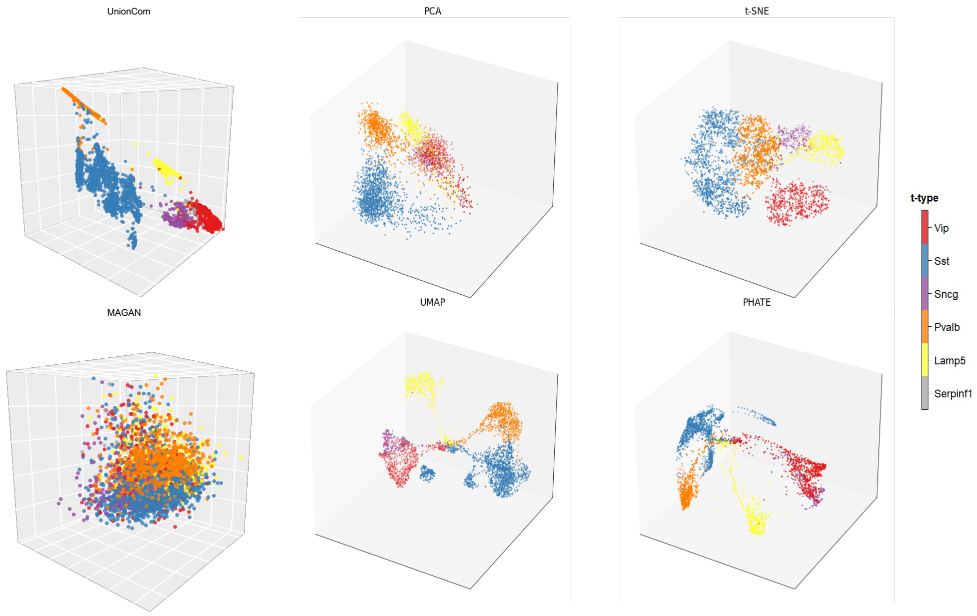

Figure S4 UnionCom, MAGAN, PCA, PHATE, t-SNE and U-map for visualizing 3,654 cells in the mouse visual cortex using their transcriptomic data. Points: cells. Colors: transcriptomic types (t-types).

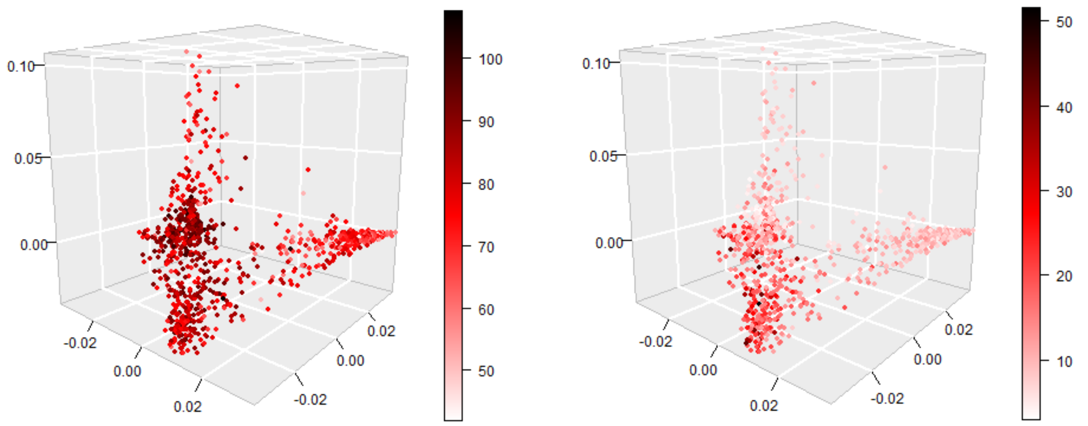

Figure S5 Continuous changes of select neuronal electrophysiological features (left, AP amplitude in mV; right, membrane time in ms) in the motor cortex. The cells are ordered by their coordinates on the latent space after nonlinear manifold alignment.

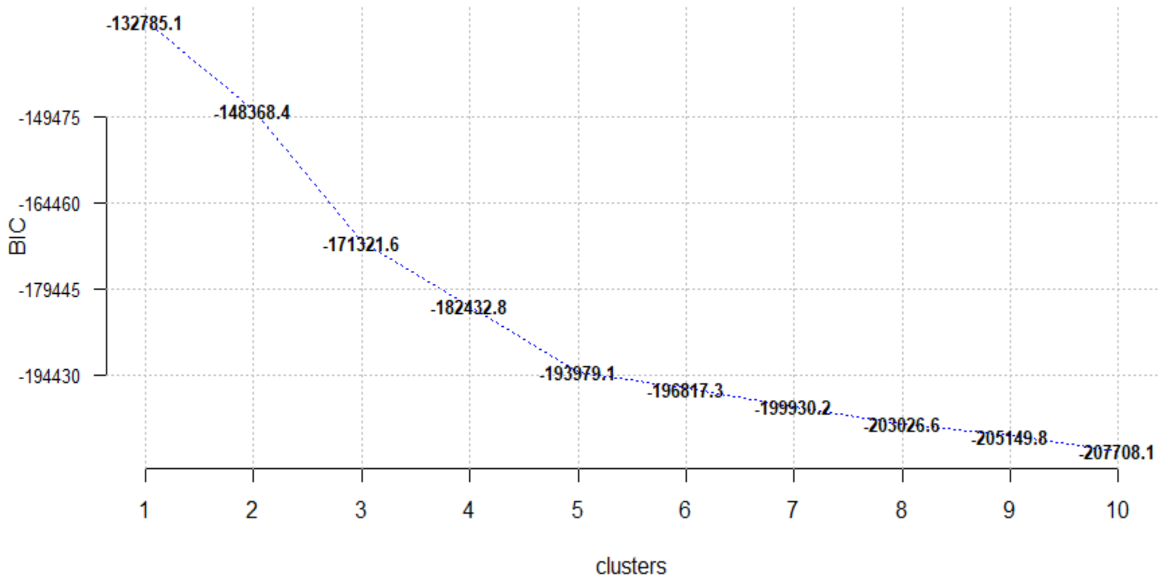

Figure S6 BIC selection result for optimal cluster numbers using gaussian mixture model, where clusters represent the total number of clusters, BIC represent the BIC value for corresponding model.

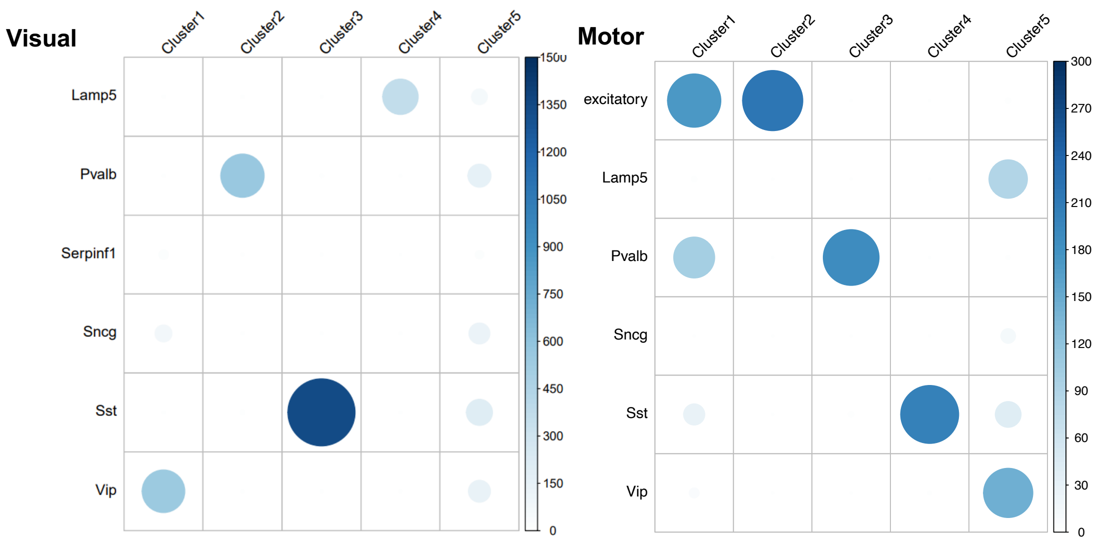

Figure S7 Comparison between t-types and the cell clusters by GMM from the latent space after nonlinear manifold alignment. (Left) mouse visual cortex. (Right) mouse motor cortex. The dot size and color in each intersection region correspond to the total number of cells they share.

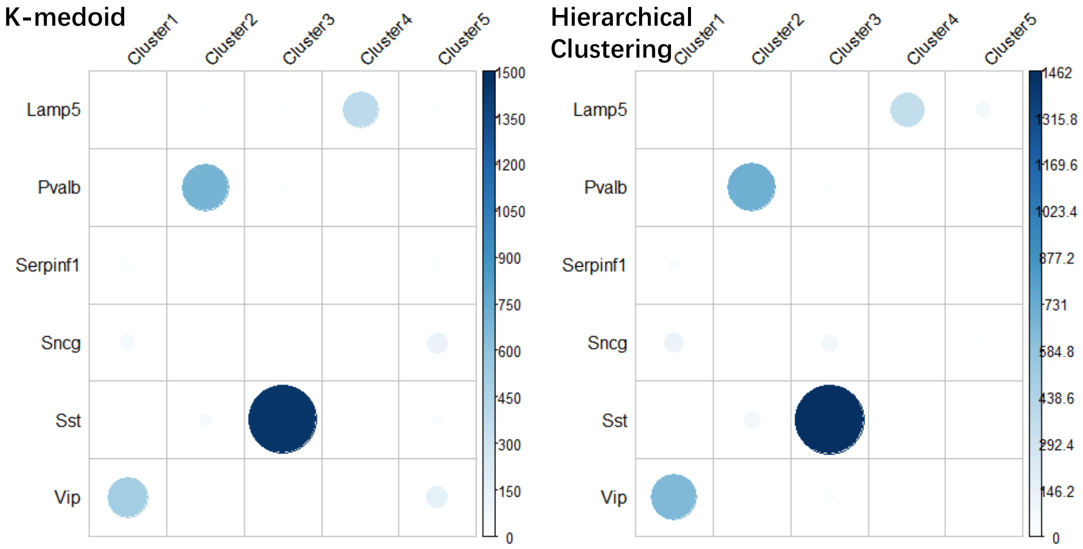

Figure S8 Numbers of overlapped cells between t-types and cross-modal cell clusters by Hierarchical Clustering (Right) and K-medoid (Left) from the latent space after nonlinear manifold alignment of single cells in the mouse visual cortex. The dot size and color correspond to the number of shared cells.

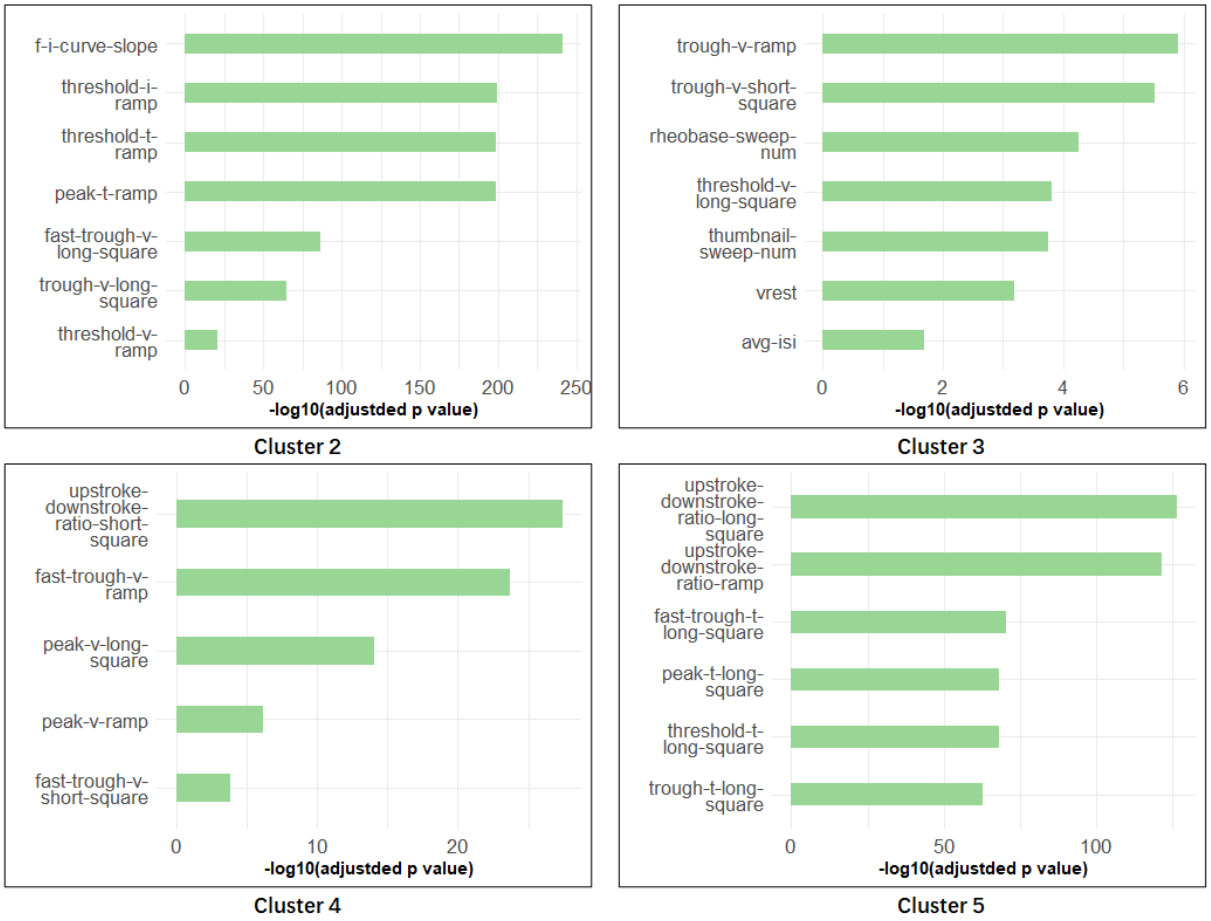

Figure S9 The representative electrophysiological features (adjusted p-value <0.05) from Cluster 2 to Cluster 5 in the visual cortex.

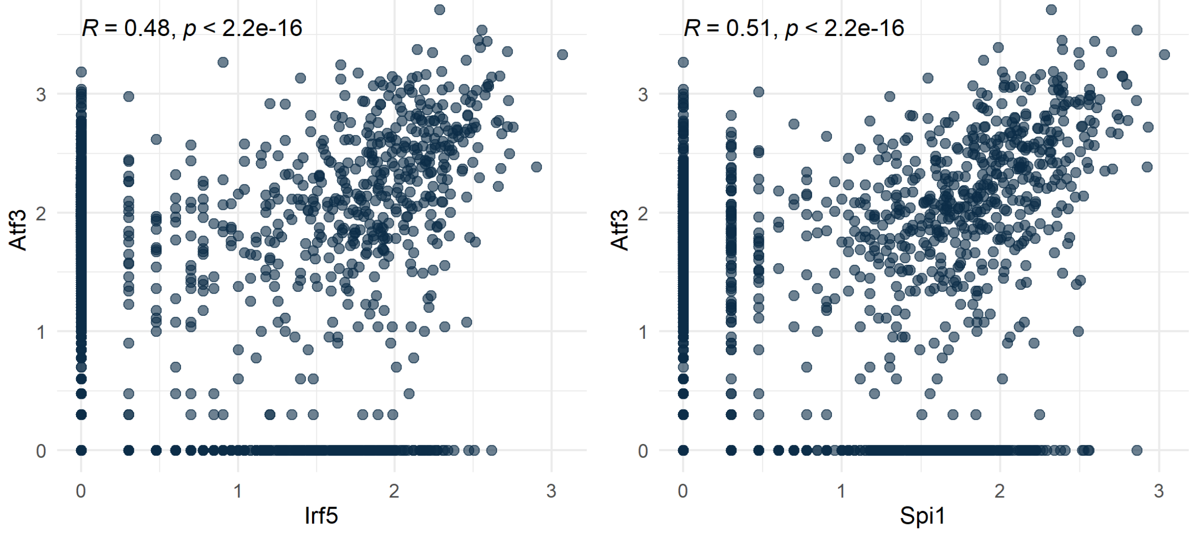

Figure S10 Gene expression levels of Atf3, Irf5 and Spi1 (immunological TFs) of cells in the mouse visual cortex. The expression level is log10 transformed.

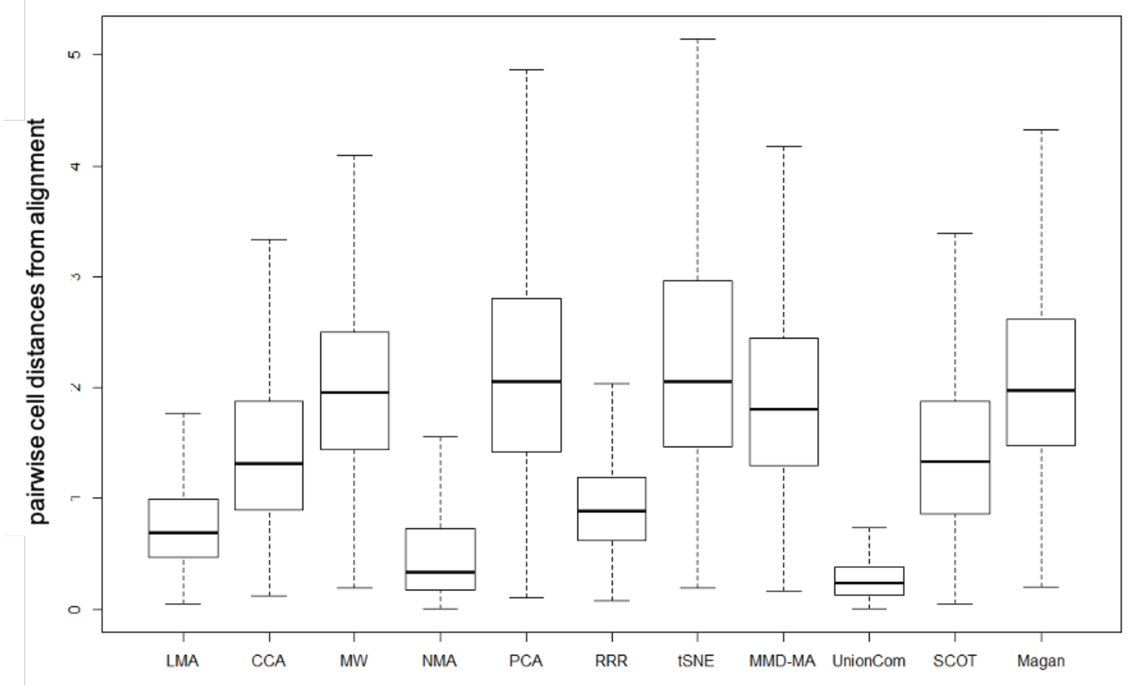

Figure S11 Boxplots show the pairwise cell Euclidean distance after 50% semi-supervised NMA alignment on the latent space for 3654 neuronal cells (aspiny) in the mouse visual cortex. Among the proposed methods, only UnionCom (average 0.280) outperforms NMA (average 0.587 distance)

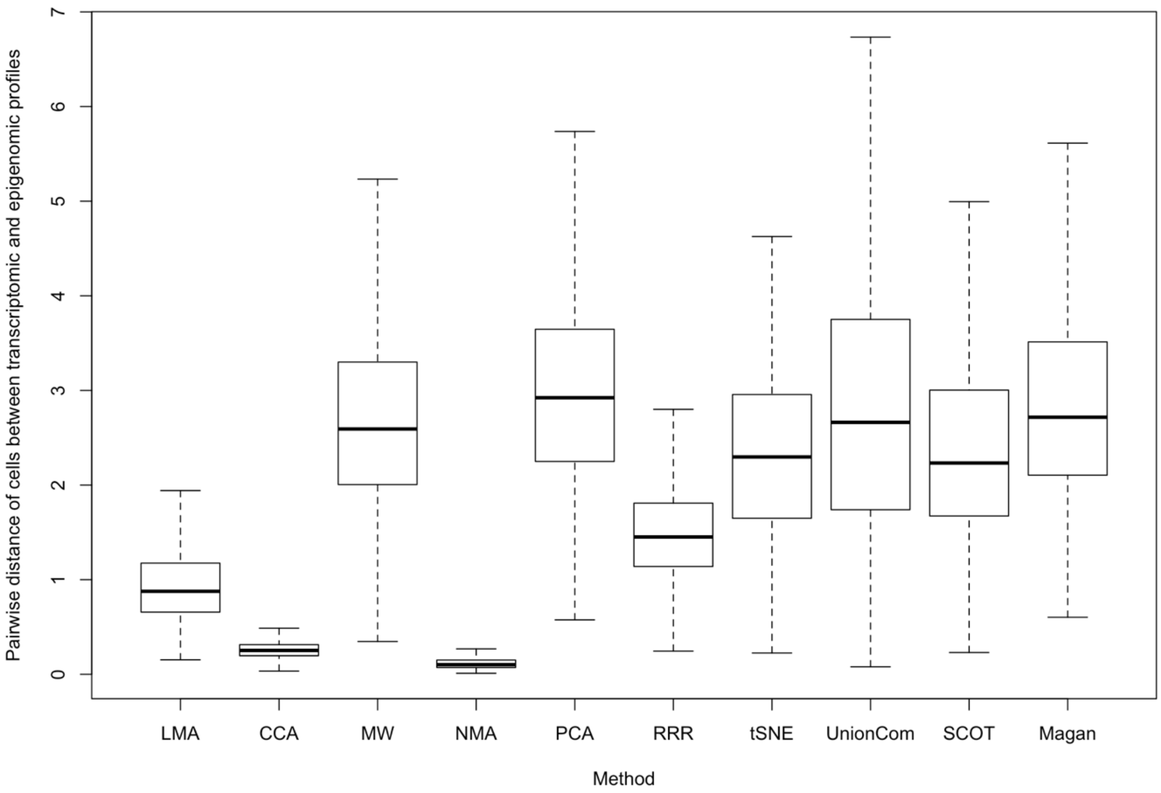

Figure S12 Pairwise Euclidean distance of aligned scATAC-seq and scRNA-seq data of 2,641 cells (HEK293T, NIH/3T3, A549 cells). The co-profiling data of scRNA-seq and scATAC-seq was generated by the assay, sci-CAR (Cao et al., Science, 2018) and downloaded from GEO: GSM3271040 and GSM3271041. The machine learning methods for alignment include linear manifold alignment (LMA), Canonical Correlation Analysis (CCA), manifold warping (MW), nonlinear manifold alignment (NMA), UnionCom, SCOT, Magan, MMD-MA, Principal Component Analysis (PCA, no alignment), and reduced rank regression (RRR), and t-Distributed Stochastic Neighbor Embedding (t-SNE, no alignment).

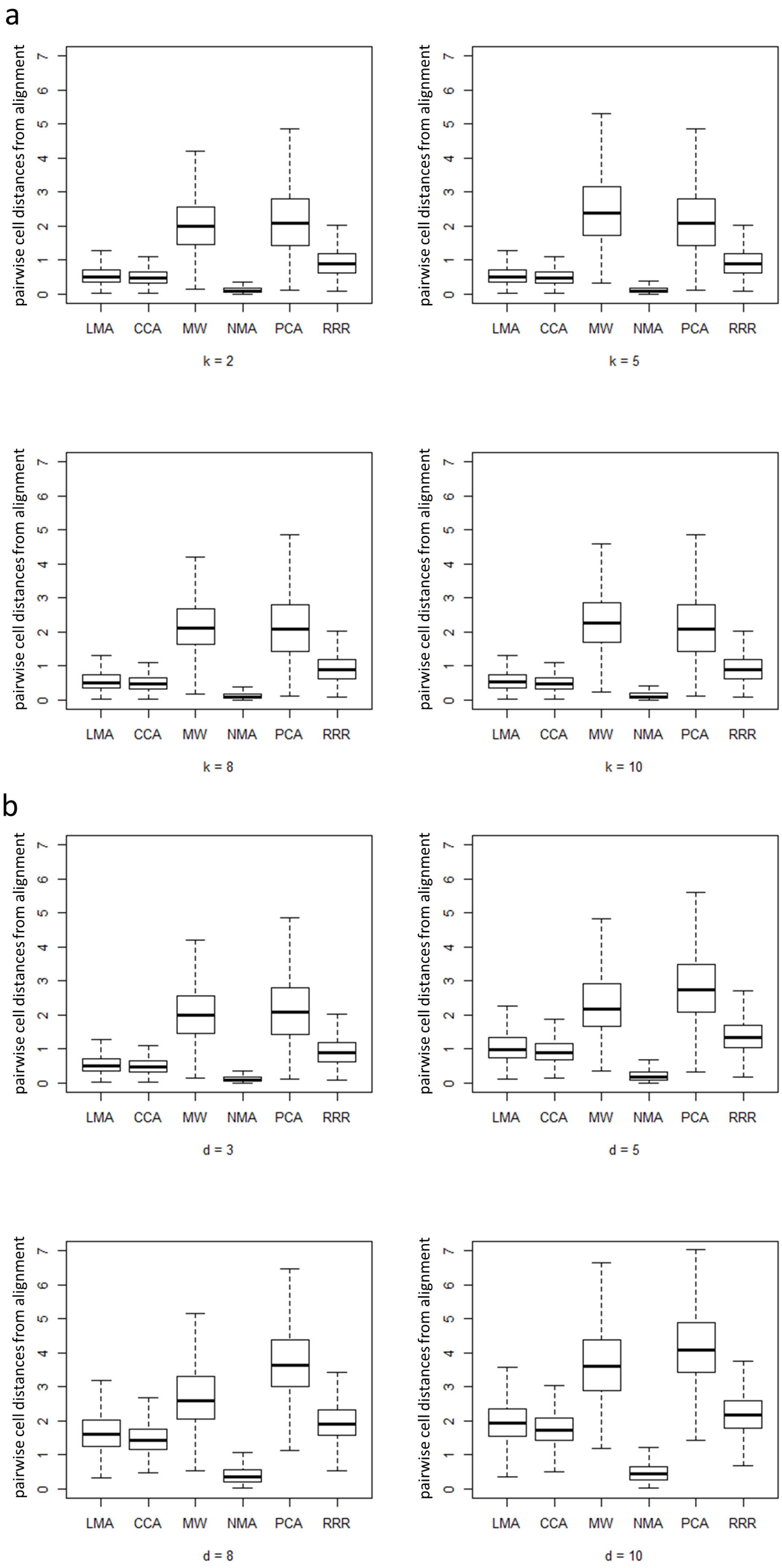
a
pairwise cell distances from alignment
LMA CCA MW NMA PCA RRR
k = 2
k = 5
k = 8
k = 10
b
d = 3
d = 5
d = 8
d = 10

Figure S13 (**a**) Boxplots show the pairwise cell distance (Euclidean Distance) after alignment on the latent space for 3654 neuronal cells in the mouse visual cortex for different choices of nearest neighbors in manifold learning methods ranging from 2 to 10 (also see Fig 1B). The cell coordinates on the latent space are standardized per cell (i.e., each row of $\tilde{X} = [\tilde{X}_e, \tilde{X}_t]$) for comparison across methods. Each box represents one alignment method. The box indicates the lower and upper quantiles of the data, with a horizontal line at the median, the vertical line extended from the boxplot shows 1.5 interquartile range beyond the 75th percentile or 25th percentile. The machine learning methods for alignment include linear manifold alignment (LM), nonlinear manifold alignment (NMA), manifold warping (MW), Canonical Correlation Analysis (CCA), and Principal Component Analysis (PCA, no alignment). (**b**) Boxplots show the pairwise cell distance (Euclidean Distance) after alignment on the latent space for 3654 neuronal cells in the mouse visual cortex for different choices of latent space dimensions ranging from 3 to 10.
