# Supplementary material for: Manifold learning analysis suggests strategies to align single-cell multimodal data of neuronal electrophysiology and transcriptomics
Source: Commun Biol. 2021 Nov 19;4:1308. doi: 10.1038/s42003-021-02807-6 (PMC8604989; doi:10.1038/s42003-021-02807-6)
Supplement: Supplementary file 3 — Description of Additional Supplementary Files [file 42003_2021_2807_MOESM3_ESM.pdf]

Description of Additional Supplementary Files

File name: Supplementary Data 1

Description: Differentially expressed genes in cell clusters

File name: Supplementary Data 2

Description: Enrichments of differentially expressed genes in cell clusters.

File name: Supplementary Data 3

Description: Gene regulatory networks for manifold-aligned cell clusters.

File name: Supplementary Data 4

Description: Gene regulatory networks for known t-types.

File name: Supplementary Data 5

Description: Testing R^2 values for electrophysiological feature prediction from gene expression.
